# How Bacterial Adaptation to Cystic Fibrosis Environment Shapes Interactions Between *Pseudomonas aeruginosa* and *Staphylococcus aureus*

**DOI:** 10.3389/fmicb.2021.617784

**Published:** 2021-03-03

**Authors:** Laura Camus, Paul Briaud, François Vandenesch, Karen Moreau

**Affiliations:** ^1^CIRI-Centre International de Recherche en Infectiologie, Université de Lyon/Inserm U1111/Université Claude Bernard Lyon 1/CNRS UMR 5308/ENS de Lyon, Lyon, France; ^2^Centre National de Référence des Staphylocoques, Hospices Civils de Lyon, Lyon, France; ^3^Institut des Agents Infectieux, Hospices Civils de Lyon, Lyon, France

**Keywords:** *P. aeruginosa*, *S. aureus*, interaction, evolution, cystic fibrosis

## Abstract

*Pseudomonas aeruginosa* and *Staphylococcus aureus* are the two most prevalent bacteria species in the lungs of cystic fibrosis (CF) patients and are associated with poor clinical outcomes. Co-infection by the two species is a frequent situation that promotes their interaction. The ability of *P. aeruginosa* to outperform *S. aureus* has been widely described, and this competitive interaction was, for a long time, the only one considered. More recently, several studies have described that the two species are able to coexist. This change in relationship is linked to the evolution of bacterial strains in the lungs. This review attempts to decipher how bacterial adaptation to the CF environment can induce a change in the type of interaction and promote coexisting interaction between *P. aeruginosa* and *S. aureus*. The impact of coexistence on the establishment and maintenance of a chronic infection will also be presented, by considering the latest research on the subject.

## Introduction

Cystic fibrosis (CF) patients suffer from severe pulmonary infections. *Pseudomonas aeruginosa* is one of the most prevalent bacteria within CF lungs and is the main factor responsible for poor clinical outcomes due to the difficulty of eradicating it. Indeed, this bacterium has been shown to rapidly adapt to the CF lung environment and persist efficiently despite host immune responses and antibiotic treatments. This adaptation is mainly due to the accumulation of genetic mutations that alter the expression profiles and phenotypes of *P. aeruginosa* (Smith et al., 2006; Marvig et al., 2015a; Winstanley et al., 2016; La Rosa et al., 2019).

Another important feature of *P. aeruginosa* is its ability to interact with other CF microorganisms such as *Aspergillus fumigatus*, *Candida albicans*, and *Staphylococcus aureus. S. aureus* is also one of the most predominant pathogens in CF lungs and is detected simultaneously with *P. aeruginosa* in 20 to 50% of patients (Limoli et al., 2016; Briaud et al., 2020). *P. aeruginosa* can exhibit particularly aggressive behavior toward *S. aureus* (Michelsen et al., 2016). This antagonistic interaction was the only one observed between the two species for a long time. However, *P. aeruginosa* strains with more tolerant behavior toward *S. aureus* have recently been isolated from chronic CF infections (Baldan et al., 2014; Michelsen et al., 2016; Briaud et al., 2019, 2020; Pallett et al., 2019; Camus et al., 2020). The establishment of such coexisting interaction between the two species seems to arise from their evolution in the lung ecosystem.

In this review, we aim to clarify the relationship between the adaptation of *P. aeruginosa* and *S. aureus* to the CF environment and the evolution of their interactions. We will present the genetic and phenotypic evolutions of the two partners and their impacts. The characterization of this coexisting interaction status and how it can contribute to the establishment and maintenance of a chronic infection will also be assessed.

## Adaptation to the CF Environment Reduces *P. aeruginosa’s* Anti-Staphylococcal Behavior

### *P. aeruginosa* Evolves During the Establishment of Chronic Infection

Lungs of CF patients constitute a stressful environment for colonizing microorganisms, especially due to toxic, osmotic, or oxidative stresses induced by the host immune system and recurring antibiotic treatments. Anoxia or micro-anaerobia, acidity and the nutritional characteristics of the CF environment can also hamper bacterial growth and persistence (Yang et al., 2011; La Rosa et al., 2019). Nevertheless, *P. aeruginosa* was shown to adapt in response to these different selective pressures. Numerous sequencing studies of longitudinal isolates of *P. aeruginosa* revealed that the bacterium accumulates a significant number of small mutations (SNP and small insertions and deletions) during its evolution in CF lungs. So, some genomic modification were found to be positively selected due to their beneficial impacts on *P. aeruginosa* fitness in the stressful CF environment (Smith et al., 2006; Marvig et al.,2015b,c; Klockgether et al., 2018; Khademi et al., 2019). There were called pathoadaptive modifications as they promote the pathogen survival and persistence at the infectious site. As a result, CF-adapted isolates of *P. aeruginosa* present convergent expression profiles and phenotypes, conferring advantageous features (Folkesson et al., 2012; Winstanley et al., 2016; La Rosa et al., 2019). Table 1 gathers the main pathoadaptive genomic regions of *P. aeruginosa*, as well as the associated phenotypes (Table 1).

**TABLE 1:** Main genetic and phenotypic adaptations of *Pseudomonas aeruginosa* during its evolution in the CF lung environment.

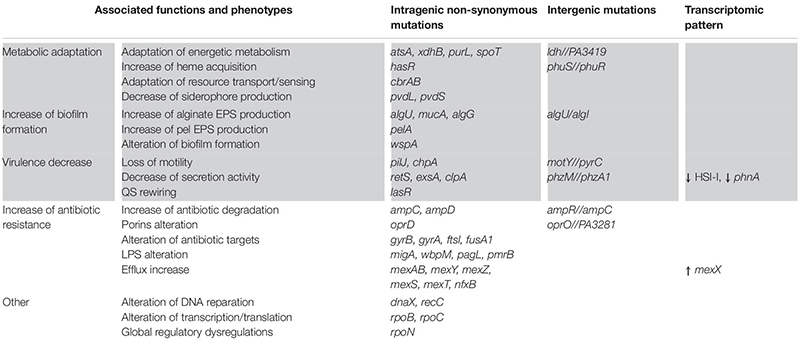

*Gray cells indicate adaptations that can impact microbial interactions of *P. aeruginosa*.*

Firstly, CF-adapted strains of *P. aeruginosa* adjust their energetic metabolism to the particular nutritional composition of the CF environment (Palmer et al., 2007; La Rosa et al., 2018, 2019). This is mainly reflected by a restriction of the catabolic repertory and the slowed growth of clinical isolates in comparison to non-adapted strains (Marvig et al., 2015a; La Rosa et al., 2018, 2019). In response to iron sequestration by the host, *P. aeruginosa* also adapts its iron uptake mechanisms for the benefit of heme-based assimilation. Alteration of *pvd* genes in CF isolates was indeed shown to decrease the production of the pyoverdine siderophore and thus ferric iron acquisition. In contrast, mutations in the *phuS//phuR* intergenic region increase the expression of the heme receptor PhuR and thus acquisition of host-sequestered iron (Table 1; Marvig et al., 2014; Minandri et al., 2016). Iron and nutrient acquisition nonetheless remains difficult in CF lung, hampering the costly production of many iron-dependent virulence factors. *P. aeruginosa* virulence is also reduced by genetic alterations and/or transcriptomic dysregulations of genomic regions involved in motility (*pilJ* and *chpA*), secretion (*phz* and *Hcp* genes) and regulation of virulence (*lasR*, *retS*, *exsA*, and *rpoN*) (Marvig et al., 2015a; Winstanley et al., 2016). Most of these factors being immunogenic, the down-regulation of their production allows a better escape of *P. aeruginosa* from the host immune system. Such escape and resistance to immune responses is also promoted by the increased biofilm production of CF-adapted *P. aeruginosa*. In particular, mutations in *mucA* and *alg* genes induce the overproduction of alginate, an exopolysaccharide composing the biofilm matrix and responsible for the frequent mucoid phenotype (Marvig et al., 2015b; Winstanley et al., 2016). Finally, adapted strains of *P. aeruginosa* exhibit increased antibiotic resistance related to genic alterations of antibiotic targets (for instance the gyrases *gyrA*, *gyrB*), efflux pumps (*mex* genes), lactamases (*amp* genes), as well as lipopolysaccharide (LPS) and porin synthesis (*pagL*, *pmrB*, and *opr* genes) (Table 1; Folkesson et al., 2012; Marvig et al., 2015b; Winstanley et al., 2016). Altogether, these adaptations induce a weakly virulent but highly resistant state of *P. aeruginosa*, promoting its persistence within CF lungs.

### Impact of *P. aeruginosa* Adaptation on Its Anti-staphylococcal Behavior

**FIGURE 1:**
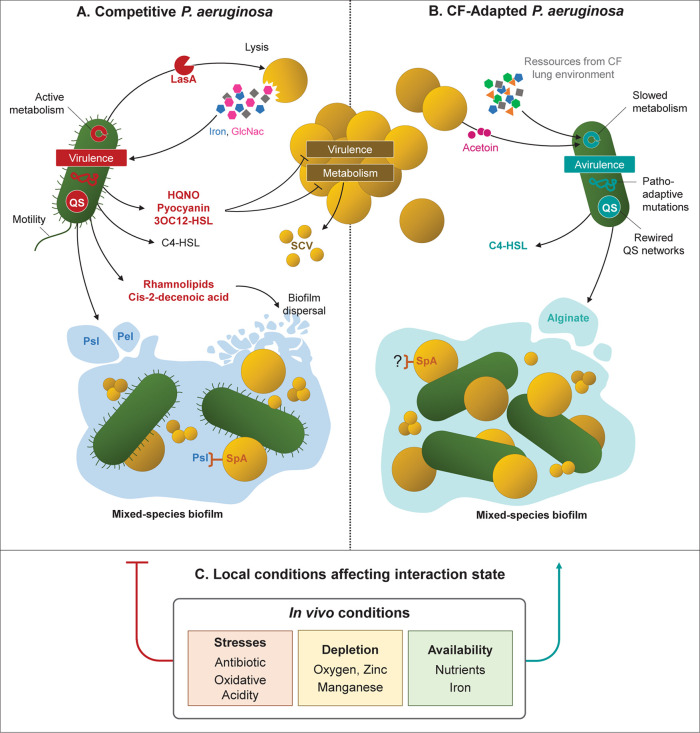
Evolution of bacterial interactions with *Staphylococcus aureus* related to *Pseudomonas aeruginosa* adaptation to CF environment. **(A)** Reference, environmental and acute infection *P. aeruginosa* isolates produce high amounts of virulence factors (LasA, HQNO, pyocyanin) or QS signals (3OC12-HSL). These factors allow *P. aeruginosa* to outcompete *S. aureus* by lysis mechanisms, growth, metabolism and virulence inhibition. Mixed-species biofilms can be formed through SpA-Psl binding, but can be disrupted by rhamnolipids and *cis-*2-decenoic acid. **(B)** *P. aeruginosa* isolates evolved in CF context coexist with *S. aureus* due to genetic and transcriptomic adaptations reducing the production of virulence and anti-staphylococcal factors. *P. aeruginosa* QS networks are rewired and regulated through the C4-HSL signal, that does not affect *S. aureus* physiology. CF-adapted isolates present a slowed and adjusted metabolism to the carbon sources of the CF environment and/or produced by *S. aureus*. Matrix of mixed-species biofilm are dominated by the alginate exopolysaccharide. **(C)** *P. aeruginosa* adaptation and its interaction with *S. aureus* are influenced by environmental factors that characterize *in vivo* conditions. Among them, antibiotic and oxidative stresses, depletion in oxygen, zinc and manganese, and high availability of nutrients and iron were shown to promote a coexisting interaction between the two pathogens. QS, quorum-sensing; SCV, small-colony variants; HQNO, 2-heptyl-4-hydroxyquinoline N-oxide; 3OC12-HSL, *N*-3-oxo-dodecanoyl homoserine lactone; C4-HSL, butyryl homoserine lactone.

In addition to limiting the effectiveness of the immune response and antibiotic treatments, adaptations of *P. aeruginosa* can also modify its interactions with other microbial species, in particular with *S. aureus*. The anti-staphylococcal behavior of *P. aeruginosa* relies on mechanisms of bacterial lysis and growth suppression, as well as metabolic alterations, virulence and biofilm formation (Figure 1A; Hotterbeekx et al., 2017). However, several studies demonstrated that such behavior was not conserved in CF-adapted strains of *P. aeruginosa*, allowing the establishment of a coexisting interaction with *S. aureus* (Baldan et al., 2014; Michelsen et al., 2014, 2016; Limoli et al., 2017; Briaud et al., 2019). It thus appears that the relationship between *P. aeruginosa* and *S. aureus* evolves from competition to coexistence, a process resulting from the adaptation of *P. aeruginosa*.

#### Nutrient Competition and Virulence

One of the main factors involved in the competitive interaction between *P. aeruginosa* and *S. aureus* is that of resources, notably iron-availability in the environment. Indeed, it has been shown that *P. aeruginosa’s* aggressive behavior toward the Gram positive bacterium varies according to medium richness and is even promoted during iron depletion (Mashburn et al., 2005; Filkins et al., 2015; Nguyen et al., 2015; Miller et al., 2017). In these conditions, *P. aeruginosa* uses *S. aureus* as an iron source by lysing its cells thanks to the LasA protease, whose production is regulated by the quorum-sensing (QS) regulator LasR (Toder et al., 1991; Mashburn et al., 2005; Hotterbeekx et al., 2017). As *P. aeruginosa’s* adaptation to CF lungs is accompanied by *lasR* mutations and QS network rewiring, protease activity and especially LasA production are frequently reduced in chronic clinical isolates (Table 1, Figure 1B; Manos et al., 2013; Marvig et al., 2015a; Feltner et al., 2016; van Mansfeld et al., 2016; O’Brien et al., 2017). The ability of *P. aeruginosa* CF strains to lyse *S. aureus* cells was recently assessed on a limited set of three isolates. It was nonetheless depicted that the isolate lacking LasA production and protease activity was the only one unable to outcompete and lyse *S. aureus* (Pallett et al., 2019). Studies including larger sets of strains with different colonization times (as longitudinal ones) would be of great interest to evaluate the relationship between *P. aeruginosa*’s adaption to the CF lung environment and its ability to efficiently lyse *S. aureus*. In addition, *S. aureus* lysis leads to increased concentrations of N-acetyl glucosamine (GlcNac) which induces the production of virulence factors by *P. aeruginosa* (Hotterbeekx et al., 2017). Thus, the absence of *S. aureus* lysis may contribute to the decrease in virulence of *P. aeruginosa*.

As a strategy to limit nutritional competition, *P. aeruginosa* is also able to inhibit the growth of *S. aureus* by altering its metabolic activities. Indeed, the secondary metabolites HQNO (2-heptyl-4-hydroxyquinoline N-oxide) and pyocyanin secreted through the PQS (Pseudomonas Quinolone Signal) system induce a fermentative metabolism in the Gram positive bacterium (Hoffman et al., 2006; Proctor et al., 2014; Hotterbeekx et al., 2017; Noto et al., 2017). This metabolic switch is also responsible for the well-described small colony variant (SCV) phenotype of *S. aureus* (Proctor et al., 2014; Figure 1A). Genes involved in HQNO synthesis (*phn* genes) are under-expressed in adapted *P. aeruginosa* isolates, whereas pyocyanin production through *phz* genes can be impacted by intergenic mutations (Table 1). As a result, adapted strains of *P. aeruginosa* frequently present reduced HQNO and pyocyanin production in comparison to early ones (Bianconi et al., 2015; Cullen et al., 2015; Michelsen et al., 2016; Limoli et al., 2017; O’Brien et al., 2017; Figure 1B). This is especially the case for isolates from the DK2 lineage, which is known for developing a proto-cooperative interaction with *S. aureus* (Michelsen et al., 2016). However, HQNO and pyocyanin production are not always reduced in CF-adapted *P. aeruginosa* since these molecules can still be detected at active concentrations within CF sputum of chronically infected patients (Wilson et al., 1988; Hoffman et al., 2006; Alatraktchi et al., 2020). In fact, O’Brien et al. (2017) observed considerable variances in pyocyanin production between strains from different patients, but also between lineages recovered from a single subject. These results suggest that the isolates evolve differently according to their niches within the lungs, leading to different metabolic activities and thus interactions with co-colonizing microorganisms. Therefore, growth inhibition of *S. aureus* would vary depending on the ability of the nearest *P. aeruginosa* bacterium to produce pyocyanin or HQNO.

In the same way, the QS signal *N*-3-oxo-dodecanoyl homoserine lactone (3OC12-HSL) was shown to inhibit *S. aureus* growth in a dose-dependent manner (Hotterbeekx et al., 2017; Figure 1A). Production of this long-chain acyl-homoserine lactone (AHL) is regulated by LasR and thus frequently reduced in *lasR*-mutated CF isolates of *P. aeruginosa.* Instead, these isolates generally maintain the production of the short-chain AHL butyryl-HSL (C4-HSL) whose production is regulated by the second QS system of *P. aeruginosa*, Rhl (Bjarnsholt et al., 2010; Feltner et al., 2016; Chen et al., 2019; Cruz et al., 2020). And interestingly, C4-HSL signals have not been shown to affect *S. aureus* (Figure 1B).

#### Mixed-Species Biofilm Formation

Another important feature of bacterial interaction is the formation of mix-biofilm. The relationship between the two pathogens within biofilms appears to be a complex situation, highly dependent on isolates and culture conditions. A first study on 63 isolates demonstrated that *S. aureus* biofilm formation was enhanced by the secondary metabolites HQNO and Alkyl Quinolones (AQs) secreted by *P. aeruginosa*. However, although the effect was clear on a specific *S. aureus* clinical strain used as control, it was less manifest on other clinical CF isolates (Fugère et al., 2014). Conversely, *S. aureus* supernatant can either stimulate or inhibit *P. aeruginosa* biofilm formation in a strain-dependent manner as well (Armbruster et al., 2016). These effects involve the surface protein A (SpA) of *S. aureus*, an immunoglobulin-binding factor responsible for immune suppression (Kobayashi and DeLeo, 2013). Besides host proteins, SpA can bind two *P. aeruginosa* structures important for biofilm formation: (i) type IV pili, involved in twitching motility, and (ii) Psl, one of the three main exopolysaccharides (EPS), with alginate and Pel, that form the extracellular matrix of *P. aeruginosa* biofilm (Leid et al., 2005; Ryder et al., 2007; Colvin et al., 2011, 2012; Armbruster et al., 2016). Chew et al. (2018) observed that Psl enables wild-type *P. aeruginosa* to outcompete *S. aureus* in early biofilm development. On the contrary, Pel is required to reduce the effective crosslinking of the matrix in late-stage biofilm development, improving super-diffusivity in microcolony regions and dual-species biofilm growth (Chew et al., 2018).

*Pseudomonas aeruginosa’s* adaptation to the CF environment affects both motility and exopolysaccharide production. As a result, chronical isolates frequently lack type IV pili and present a biofilm matrix dominated by alginate, especially in the mucoid phenotype (Høiby et al., 2017). Therefore, one might wonder if the modulation of mixed-species biofilm formation through Pel, Psl, and SpA occur in adapted CF isolates in the same way as for reference strains (Figure 1B).

Competitive *P. aeruginosa* are also able to disperse *S. aureus* biofilm or limit its establishment through the secretion of rhamnolipids and *cis-*2-decenoic acid (Hotterbeekx et al., 2017; Figure 1A). To our knowledge, the production of *cis-*2-decenoic acid was not assessed in CF-adapted clinical *P. aeruginosa*, but rhamnolipid synthesis has been studied more extensively. These molecules induce significant inflammatory host responses, promote *P. aeruginosa* motility, and their synthesis is favored during planktonic growth (Déziel et al., 2003; Alhede et al., 2014; Hotterbeekx et al., 2017). As these phenotypes are frequently reduced in chronic *P. aeruginosa* isolates, it therefore seems likely that bacterial adaptation to the CF lung is accompanied by a decrease of rhamnolipid production. Indeed, chronic isolates studied by Bjarnsholt et al., and the CF-adapted lineage DK2 were shown to produce less rhamnolipids than intermittent and reference *P. aeruginosa* strains (Bjarnsholt et al., 2010; Michelsen et al., 2016). Low rhamnolipid synthesis was also recently associated with alginate overproduction and the common mucoid phenotype of *P. aeruginosa*. The presence of alginate, either produced by *mucA*-mutated mucoid strains or exogenously added in the medium, induced transcriptomic and post-transcriptomic down-regulation of rhamnolipid synthesis (Limoli et al., 2017; Price et al., 2020). Alginate-producing *P. aeruginosa* also presented reduced ability to outcompete *S. aureus* (Limoli et al., 2017; Price et al., 2020). Altogether, this suggests that decreased rhamnolipid production and increased alginate synthesis in adapted *P. aeruginosa* isolates contribute to improve *S. aureus* survival during mixed-biofilm formation (Figure 1B). In connection with this, Baldan et al. (2014) showed that early and late CF *P. aeruginosa* isolates presented different behaviors when grown in biofilm with *S. aureus*. This latter exhibited better viability with the CF-adapted isolate of *P. aeruginosa* and was also able to alter its biofilm production (Baldan et al., 2014).

### Impact of Environmental Factors on the Anti-staphylococcal Behavior of *P. aeruginosa*

Although bacterial features play an important role in shaping inter-species interaction, environmental factors can have an even more decisive impact on them. Environmental conditions such as antibiotic and oxidant stresses are known to shape the CF ecosystem and promote a biofilm-based lifestyle of microorganisms within lungs (Feraco et al., 2016; Riquelme et al., 2020). Biofilm conditions reducing the competitive relationship between *P. aeruginosa* and *S. aureus*, the CF environment may favor a coexistence interaction between the two pathogens independently of *P. aeruginosa’s* genetic adaptation. Indeed, several environmental factors inherent to CF lungs were shown to decrease the anti-staphylococcal behavior of *P. aeruginosa*. Pallett et al. (2019) recently observed that oxygen limitation decreased the production of *P. aeruginosa* virulence factors such as proteases and pyocyanin. In these conditions, not only the reference strain PAO1 but also clinical isolates thus coexisted with *S. aureus* despite competitive behavior under normoxia (Pallett et al., 2019). Within mixed-species biofilm formed with the PA14 reference strain, *S. aureus* survival was improved thanks to bacterial stratification as a function of oxygen levels (Cendra et al., 2019). In addition to oxygen, nutrient availability can also modify interaction within biofilm (Mashburn et al., 2005; Filkins et al., 2015; Nguyen et al., 2015; Hotterbeekx et al., 2017; Miller et al., 2017; Cendra et al., 2019). Miller et al. (2017) observed that *S. aureus* was even able to strongly outcompete *P. aeruginosa* within mixed-species biofilm grown under rich conditions, but not in the same diluted and thus impoverished medium. Besides nutritive richness, limited alkalization of the co-culture medium was also shown to improve *S. aureus* survival during interaction with the PA14 reference strain (Cendra et al., 2019). Finally, the depletion of zinc and manganese, notably observed on the edge of the biofilm architecture, represses the expression of *P. aeruginosa’s* virulence genes and thus *S. aureus* inhibition (Wakeman et al., 2016; Figure 1C).

Interestingly, all these conditions promoting coexistence interaction between the two pathogens seem to be combined in the CF environment. CF sputum is indeed considered a rich medium but it contains limited oxygen concentrations, favoring anaerobic and micro-anaerobic metabolisms (La Rosa et al., 2019). Sputa from CF patients were also shown to be relatively acidic (Bhagirath et al., 2016). Moreover, host-pathogen interfaces are known to be depleted in zinc and manganese, especially through sequestration by the host protein calprotectin (Kehl-Fie and Skaar, 2010; Hood et al., 2012; Wakeman et al., 2016). Altogether, this suggests that *in vivo* conditions, and especially the CF environment, may promote non-competitive interaction between *P. aeruginosa* and *S. aureus* (Figure 1C). This hypothesis is supported by several studies showing the improved survival of *S. aureus* during non-human *in vivo* co-infections with *P. aeruginosa*, in comparison to *in vitro* co-cultures. Yadav and colleagues demonstrated a higher proportion of *S. aureus* within an *in vivo* mixed-species biofilm in the presence of *P. aeruginosa* (Yadav et al., 2017; Millette et al., 2019). In addition, *P. aeruginosa* strains such as PA14 were shown to promote *S. aureus* colonization and maintenance in a murine lung infection model (Yadav et al., 2017; Millette et al., 2019). Similar results were obtained in the context of chronic wound co-infections (Pastar et al., 2013; DeLeon et al., 2014). Since these co-infections were maintained for a limited duration (24 h to 1 week), it is unlikely that genetic adaptation drove the establishment of a coexistence interaction between *P. aeruginosa* and *S. aureus*. This phenomenon seems to be more related to non-fixed acclimatization reducing *P. aeruginosa* virulence, or spatial compartmentalization isolating the two species from each other *in vivo*. In all cases, it appears that besides promoting *P. aeruginosa* genetic adaptation and then reducing anti-staphylococcal behavior, *in vivo* conditions are themselves favorable to non-competitive interactions with *S. aureus* (Figure 1C).

## *S. aureus* Adaptation to the CF Environment Can Also Impact Its Interaction With *P. aeruginosa*

The relationship between the two pathogens was shown to rely solely on *P. aeruginosa* anti-staphylococcal factors, whose production evolves during its adaptation and allows steady coexistence with *S. aureus*. However, although less studied, the adaptive mechanisms of *S. aureus* also promote its long-term persistence within CF lungs and may impact its interactions with co-colonizing *P. aeruginosa* strains.

### *S. aureus* Adaptation to the CF Environment

**FIGURE 2:**
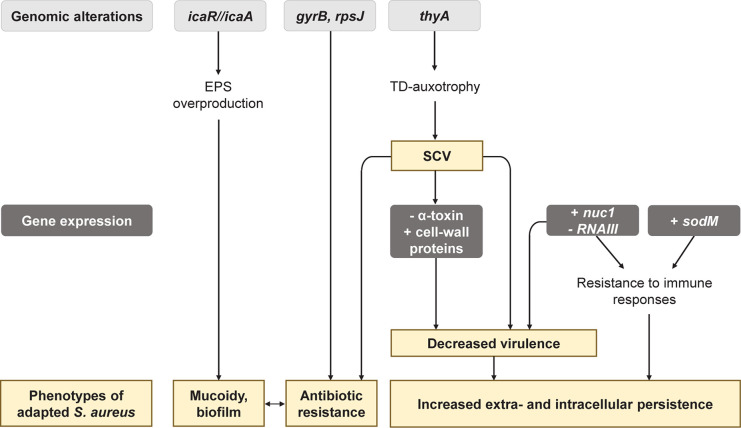
Main genetic and phenotypic adaptations of *Staphylococcus aureus* during its evolution in the CF lung environment. EPS, exopolysaccharide; TD, thymidine; SCV, small colony variants; NETs, Neutrophil Extracellular Traps.

A 21-month longitudinal study performed on 183 CF patients depicted that the clonal diversity of *S. aureus* strains decreased as patient aged, pointing out that some isolates tended to adapt more efficiently and dominate the airway sphere during infection history (Westphal et al., 2020). Several studies indeed highlighted that *S. aureus* isolates evolve in the CF lung environment and acquire features leading to better-fitted isolates (Figure 2; Chatterjee et al., 2008; Treffon et al., 2018, 2020; Herzog et al., 2019; Lennartz et al., 2019; Tan et al., 2019; Westphal et al., 2020).

Genomic, transcriptomic, and proteomic comparisons between early and late strains of *S. aureus* highlighted adaptations in the transport and the metabolism of carbohydrates and amino acids (Treffon et al., 2018; Tan et al., 2019). Late isolates thus often presented auxotrophies toward thymidine (TD), menadione or hemin (Kahl et al., 1998). These auxotrophies promote the formation of SCV (Chatterjee et al., 2008; Tan et al., 2019), a frequent *S. aureus* phenotype isolated from 4 to 50% of CF patients depending on the studies (Kahl et al., 2016). *S. aureus* SCVs are associated with worse lung function and patient age, in relation with their increased antibiotic resistance and persistence (Besier et al., 2007). Indeed, mutations in the *thyA* gene hindering TD biosynthesis were shown to facilitate resistance to trimethoprim-sulfamethoxazole (SXT) and the presence of TD-auxotrophic SCV is associated with previous antibiotic treatment with SXT (Kahl et al., 1998; Chatterjee et al., 2008; Yagci et al., 2013; Figure 2). Late isolates from antibiotic-treated patients showed other mutations in *gyrB* and *rpsJ*, that are not associated with the SCV phenotype but can explain antibiotic resistance to fluoroquinolones and cyclines (Kriegeskorte et al., 2015; Tan et al., 2019; Figure 2). Besides antibiotic resistance, SCVs are specialized for extracellular and intracellular persistence. This phenomenon is related to: (i) decreased expression of the α-toxin involved in lysis of eukaryotic cells by *S. aureus*, and (ii) increased expression of cell wall-associated genes which facilitate colonization to extracellular matrix proteins and internalization in eukaryotic cells (Figure 2). In line with this, *S. aureus* SCVs were shown to survive better intracellularly in eukaryotic cells than their isogenic parental strain (Kahl et al., 2016). CF-adapted *S. aureus* strains also tend to be better intracellular survivors than the corresponding early isolates (Treffon et al., 2020).

Another feature promoting the persistence of *S. aureus* CF isolates is the improvement of defense mechanisms against killing by polymorphonuclear leukocytes. Neutrophils usually eradicate invading pathogens by releasing reactive oxygen species (ROS) or forming Neutrophil Extracellular Trap (NETs). In comparison to their early counterparts, CF-adapted *S. aureus* presented an increased genic expression of the superoxide dismutase SodM involved in ROS detoxification (Treffon et al., 2018, 2020). Moreover, Herzog et al. (2019) showed that late isolates were more resistant to NETs mediated-killing through the over-expression of the *nuc1* gene encoding the nuclease protein. Given the high pro-inflammatory territory in patient lungs overwhelmed by the neutrophilic attacks, the over-production of SodM and Nuc1 could be a major step in the adaptation of *S. aureus* in CF lungs (Figure 2).

Interestingly, *S. aureus* isolates overexpressing the *nuc1* gene also presented a down-expression of the RNA regulator RNAIII. RNAIII is the major effector of the *agr* QS-system of *S. aureus* that positively controls the expression of many virulence factors. A decrease of RNAIII expression thus reflects reduced production of virulence factors. *nuc1*-overexpressing *S. aureus* were also shown to overexpress protein A (SpA), permease, coagulase, and adhesins (Jenul and Horswill, 2018), involved in colonization. Similarly, transcriptional analysis revealed that *S. aureus* SCVs have a less virulent phenotype in comparison to normal isolates (Kahl et al., 2005; Moisan et al., 2006; Seggewiss et al., 2006). Altogether, these results suggest that adaptation to the CF environment could lead toward a low-virulence but highly invasive state of *S. aureus*.

Finally, biofilm production appears to be increased in late *S. aureus* isolates. In the study of Tan et al. (2019), two out of three CF-late isolates over-produced biofilm and the same pattern was observed within late isolates of non-CF lung infections, suggesting that this characteristic was independent of the CF-lung environment. Such increased biofilm production by late isolates can be related to the development of mucoidy in *S. aureus*. This phenotype arises from a 5 bp deletion within the intergenic region of *icaR/icaA* genes, inducing an overproduction of *S. aureus* exopolysaccharide poly-*N*-acetylglucosamine (Schwartbeck et al., 2016; Figure 2). Mucoid *S. aureus* isolates are thus found in 2.5% of CF airways and tend to be more frequently isolated from older patients than non-mucoid isolates (Lennartz et al., 2019). These data suggest that mucoidy and strong biofilm production play a role in *S. aureus* adaptation to the CF environment.

### Impact of *S. aureus* Adaptation on Its Interaction With *P. aeruginosa*

Some of the *S. aureus* adaptations to the CF environment can impact its relationship with *P. aeruginosa*. Among them, the SCV phenotype appears to be crucial for *S. aureus* survival during competitive interaction, as SCV present increased resistance to *P. aeruginosa* mediated-killing (Hotterbeekx et al., 2017). Once *P. aeruginosa* has evolved to a non-aggressive status toward *S. aureus*, the staphylococcus can then switch to a non-defective growth mode and coexist with its partner. The increased biofilm production of CF-adapted *S. aureus* isolates could also promote the formation of mixed-species biofilm with *P. aeruginosa*, although no direct correlation has yet been established. These hypotheses could explain (i) the high proportion of co-infected patients with *S. aureus* and *P. aeruginosa* in international cohorts (Hubert et al., 2013; Limoli et al., 2016; Briaud et al., 2020), and (ii) the high proportion of isolates presenting a non-competitive interaction (Briaud et al., 2020). Further investigations need to be conducted to confirm this model and fully understand the effects of *S. aureus* adaptation on its relationship with *P. aeruginosa*.

However, several studies suggested that *S. aureus* can influence the establishment of a coexisting interaction through alterations of the adaptive process of *P. aeruginosa*. Using an *in vitro* evolution assay, the adaptation of *P. aeruginosa* was studied in the presence or absence of *S. aureus* over 150 generations (Tognon et al., 2017). Mutations in the LPS biosynthesis pathway occurred only in the presence of *S. aureus* and increased *P. aeruginosa’s* fitness and resistance toward β-lactams antibiotics (Tognon et al., 2017). Repeated *in vitro* co-cultures with *S. aureus* also induced a decrease of *P. aeruginosa* QS regulation and may provide a departure point for a coexisting interaction (Zhao et al., 2018). It is worth recalling that modifications of LPS production, increased antibiotic resistance and down-regulation of QS are also frequently depicted in CF-adapted *P. aeruginosa* isolates (Folkesson et al., 2012; Marvig et al., 2015b; Winstanley et al., 2016). These results indicate that the presence of *S. aureus* influences *P. aeruginosa’s* adaptation, and that a co-evolution phenomenon could even promote a non-competitive relationship between the two pathogens. Several results obtained from CF clinical strains support these hypotheses. First, Martha et al. (2010) observed that *P. aeruginosa* presented a higher probability to develop a mucoid phenotype in the absence of *S. aureus*. Secondly, coexisting strains of *S. aureus* and *P. aeruginosa* were shown to better produce and catabolize acetoin, respectively, in comparison to competitive strains. The authors concluded that strains co-evolved to promote trophic cooperation (Camus et al., 2020). Finally, the genetic alterations of LasR that reduce *P. aeruginosa* anti-staphylococcal behavior are frequently observed in longitudinal studies (Marvig et al.,2015b,c). One might wonder if the presence of *S. aureus* in CF lungs could contribute to the selection of *lasR* mutants and thus non-competitive *P. aeruginosa*. Indeed, promoting a coexisting and even a cooperative behavior with other co-colonizing microorganisms can be a strategy to improve fitness in a stressful environment such as CF lungs. However, most of longitudinal studies focused on *P. aeruginosa* and no data are available concerning potential co-infections with *S. aureus*. It thus remains difficult to determine if *S. aureus* can effectively influence the fixation of *lasR* mutations in *P. aeruginosa* genome. It would be interesting to study the long-term evolution of both *S. aureus* and *P. aeruginosa* in several co-infected CF patients to reveal the bacterial adaptations leading to the establishment of coexistence.

## Coexistence Promotes Cooperative Behaviors Between *P. aeruginosa* and *S. aureus*

### Characterization of the Coexistence Interaction Status

**FIGURE 3:**
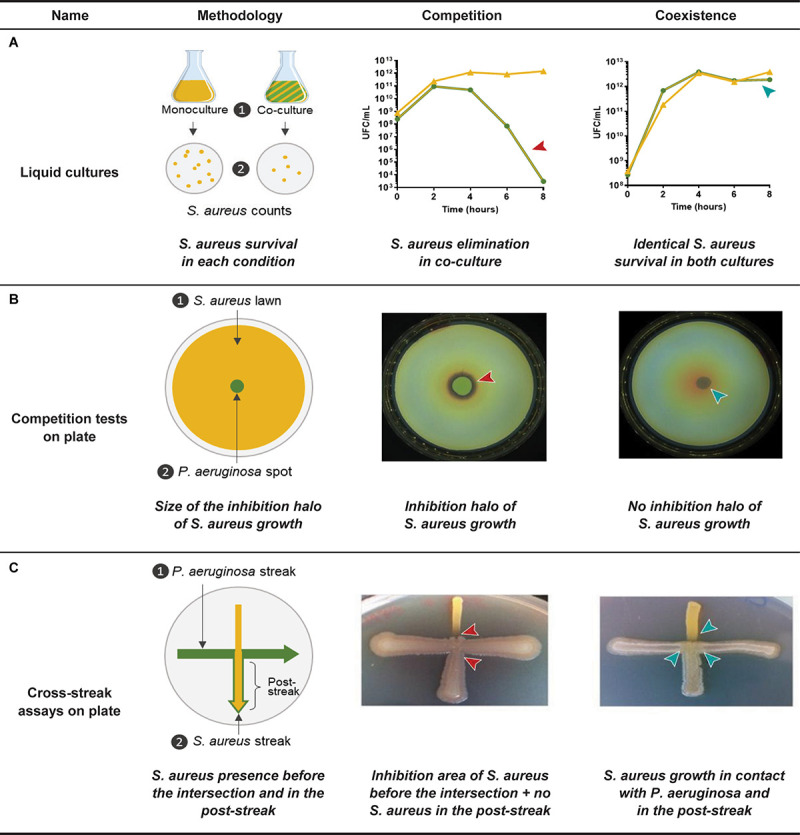
Identification methods of the coexisting interaction between *Pseudomonas aeruginosa* and *Staphylococcus aureus*. **(A)** *P. aeruginosa* and *S. aureus* are cultivated in liquid mono- and co-culture in a rich medium during 8-h and *S. aureus* cells are counted in each condition. Competition is characterized by a rapid elimination of *S. aureus* in co-culture in comparison to the monoculture, whereas *S. aureus* growth is not affected during the whole kinetic in the case of a coexisting interaction (Briaud et al., 2019; Camus et al., 2020). **(B)** *P. aeruginosa* and *S. aureus* suspensions are prepared from overnight precultures in a rich medium and *S. aureus* lawn is uniformly plated on a rich agar medium. A *P. aeruginosa* spot is deposited in the center of the lawn and the plate is incubated 24 h. Competition is characterized by an inhibition halo of *S. aureus* growth in contact with the spot of *P. aeruginosa*, whereas *S. aureus* growth is not affected by *P. aeruginosa* during the coexisting interaction. Size of the inhibition halo can be measured (Baldan et al., 2014; Briaud et al., 2019, 2020; Camus et al., 2020). **(C)** *P. aeruginosa* streak is performed on a rich agar medium, after what a perpendicular *S. aureus* streak is added. The plate is incubated during 24 h. Competition is characterized by an inhibition of *S. aureus* growth in contact with the streak of *P. aeruginosa* and a low *S. aureus* proportion in the post-streak. Coexistence is characterized by a *S. aureus* growth in contact with the streak of *P. aeruginosa* and a high *S. aureus* proportion in the post-streak. Both of these parameters are visually determined (Michelsen et al., 2016; Minandri et al., 2016; Hotterbeekx et al., 2017). Arrows indicate when or where the competitive (red arrow) or coexisting (blue arrow) interaction is observed.

Co-existence interaction leads to increased *S. aureus* survival in comparison to a common competitive interaction with *P. aeruginosa*. Bacterial enumerations performed during co-culture kinetics can thus easily reveal and quantify this phenomenon (Miller et al., 2017; Cendra et al., 2019; Pallett et al., 2019; Figure 3A). Less cumbersome procedures were nonetheless developed to assess interaction status between clinical CF isolates in large set of strains (Figure 3B; Baldan et al., 2014; Michelsen et al., 2014, 2016; Limoli et al., 2017; Briaud et al., 2019, 2020; Camus et al., 2020). Baldan and colleagues thus performed competition tests on plates during which a spot of *P. aeruginosa* culture is deposited on a lawn of *S. aureus*. Four categories of interaction status were established (no, weak, strong, and very strong inhibition) according to the size of the inhibition halo of *S. aureus* induced by the 24 *P. aeruginosa* strains tested (Baldan et al., 2014). In other studies, growth inhibition of *S. aureus* was observed through cross-streak assays and visual evaluation of each bacterium population after the streak intersection (Michelsen et al., 2014, 2016; Limoli et al., 2017; Figure 3C).

Despite the different methods and media employed, several studies highlighted that the interaction pattern with *S. aureus* was related to the adaptation of *P. aeruginosa* to the CF environment. Reduced anti-staphylococcal behavior was indeed observed for late isolates of *P. aeruginosa*, which were recovered several years after their clonal ancestor and presented common pathoadaptive traits. This evolution was thus shown in the DK2 lineage as well as in mucoid isolates (Michelsen et al., 2014, 2016; Limoli et al., 2017). However, it is noteworthy that the adaptation of *P. aeruginosa* in the CF lung is driven by adaptive radiation and spatial isolation mechanisms, inducing a high genotypic and phenotypic heterogeneity in the evolving population (Winstanley et al., 2016). It is therefore likely that clonally related isolates simultaneously recovered from the same patient can present different interaction statuses with *S. aureus* due to such diversification processes. On the other hand, it is also conceivable that the interaction statuses of competition and coexistence are dynamic and transitional within a single *P. aeruginosa* population. Assessing the interaction status of numerous *P. aeruginosa* isolates, collected longitudinally or recovered from different pulmonary niches, would lead to better understanding of this phenomenon.

**FIGURE 4:**
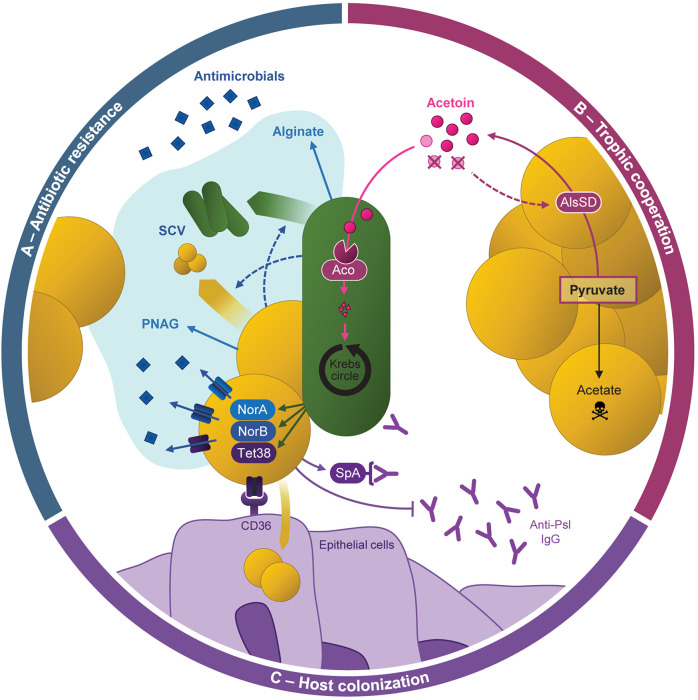
Cooperative behaviors between *Pseudomonas aeruginosa* and *Staphylococcus aureus* during coexistence. **(A)** Antibiotic resistance of both pathogens can be increased by the formation of mixed-species biofilm, especially through the production of alginate and poly-*N*-acetylglucosamine (PNAG) by *P. aeruginosa* and *S. aureus*, respectively. *P. aeruginosa* and *S. aureus* formation of small-colony variants (SCV) is also promoted during their interaction. *P. aeruginosa* presence was also shown to induce the over-expression of efflux pumps from the Nor family in *S. aureus*, enhancing its antibiotic resistance. **(B)** *S. aureus* produces acetoin from pyruvate thanks to AlsSD. *P. aeruginosa* catabolizes the acetoin produced by *S. aureus* and uses it as an alternative carbon source thanks to the *aco* system to feed the Krebs cycle. It improves its growth during co-culture with *S. aureus*. This catabolism also increases *S. aureus* survival potentially through a feed-back on acetoin production: the medium depletion in acetoin would promote AlsSD activity, limiting acetate production from pyruvate and thus cell acidification. **(C)** Resistance to immune system. *P. aeruginosa*-induced overexpression of *tet38* improves *S. aureus* internalization in epithelial cells, allowing to hide from the immune system. *S. aureus* can limit *P. aeruginosa*-induced immune responses, notably through the binding of *S. aureus* protein A (SpA) to anti-*P. aeruginosa* antibodies (Anti-Psl IgG).

In all cases, coexistence could be defined as a neutral interaction during which both bacteria are not affected by each other. However, increasing evidence shows that cooperative behaviors can positively affect *P. aeruginosa* and *S. aureus* during coexistence (Figure 4).

### Increased Resistance to Antimicrobial Compounds

Several *in vitro* studies focusing on CF isolates highlighted that coexistence between *P. aeruginosa* and *S. aureus* can improve the antibiotic resistance of both pathogens (Figure 4A). *S. aureus* was recently shown to overexpress genes coding efflux pumps from the Nor family during coexisting interaction with *P. aeruginosa*. As a result, clinical *S. aureus* isolates presented significantly increased resistance to tetracycline and ciprofloxacin in the presence of *P. aeruginosa*, in comparison to monocultures (Briaud et al., 2019). While direct contact between bacterial cells was presumed to mediate this effect, other studies identified *P. aeruginosa* secreted products affecting the resistance of *S. aureus* to antimicrobial compounds. In particular, secondary metabolites from the 4-hydroxy-2-alkylquinolines (HAQ) family such as HQNO increase the resistance of *S. aureus* to aminoglycosides notably through the induction of the SCV phenotype i (Hoffman et al., 2006; Mitchell et al., 2010; DeLeon et al., 2014; Orazi and O’Toole, 2017; Orazi et al., 2019, 2020). Interestingly, late *P. aeruginosa* isolates from CF infections often lack HQNO production but are still able to protect *S. aureus* from tobramycin. The implication of other molecules than HQNO was confirmed by an atypical HAQ profile developed during adaptation to the CF environment (Michelsen et al., 2016). It thus appears that both competitive (i.e., HQNO-producing) and coexisting *P. aeruginosa* can protect *S. aureus* from antibiotic effects but through different mechanisms. Alginate produced by CF-adapted *P. aeruginosa* may also protect *S. aureus* from antimicrobial compounds since this exopolysaccharide can impact HAQ synthesis and mixed-species biofilm formation (Limoli et al., 2017; Price et al., 2020).

Conversely, interaction with *S. aureus* can also trigger resistance mechanisms in *P. aeruginosa* (Figure 4A). Co-evolution with *S. aureus* was shown to promote the alteration of genes from the LPS biosynthesis pathway in PAO1, leading to greater resistance to β-lactam antibiotics (Tognon et al., 2017). Michelsen and colleagues demonstrated that *S. aureus* cells and their supernatant can also induce the SCV phenotype and antibiotic tolerance in CF-adapted strains *of P. aeruginosa* (Fugère et al., 2014; Michelsen et al., 2014). Similar results were obtained using persistent CF isolates of *P. aeruginosa* grown in biofilm, as they presented improved tobramycin resistance upon exposure to *S. aureus* supernatant. In this case, the phenomenon was attributed to the formation of *P. aeruginosa* aggregates within the biofilm architecture (Beaudoin et al., 2017).

### Development of Trophic Cooperation

Recent transcriptomic studies suggested that trophic cooperation could establish between *S. aureus* and *P. aeruginosa* (Figure 4B). The four-carbon molecule acetoin, produced by *S. aureus*, was shown to induce the overexpression of the *aco* system in CF *P. aeruginosa* strains during coexistence interaction (Camus et al., 2020). In line with this, *P. aeruginosa* presented an enhanced ability to catabolize acetoin produced by *S. aureus* as an alternative carbon source, resulting in increased survival during co-culture. Acetoin catabolism was also shown to benefit *S. aureus* and improve its survival in co-culture. Acetoin thus appears to be the keystone of trophic cooperation between *P. aeruginosa* and *S. aureus* during which both partners are beneficiaries. Interestingly, this cooperative behavior seems to be selected during bacterial evolution in the CF environment. Acetoin production by *S. aureus* and catabolism by *P. aeruginosa* were indeed more efficient for coexisting isolates in comparison to competitive ones (Camus et al., 2020). As acetoin could be detected in CF sputa, these results go along with the trophic adaptation of *P. aeruginosa* strains to resources present in the CF environment (Španěl et al., 2016; La Rosa et al., 2018, 2019; Camus et al., 2020). Other studies suggest that lactate, also detected in CF sputa, may also play a role in metabolic interactions between *P. aeruginosa* and *S. aureus* (Filkins et al., 2015; Tognon et al., 2017; La Rosa et al., 2019). Co-culture with *P. aeruginosa* was shown to induce the overexpression of genes involved in glucose fermentation and lactate production in *S. aureus*, leading to lactate accumulation in the medium. In turn, *P. aeruginosa* overexpressed genes responsible for lactate utilization and was able to use the molecule as a carbon source (Filkins et al., 2015; Tognon et al., 2017). However, this phenomenon was observed during a competitive interaction (using PAO1 or PA14 strains) and the impacts of lactate catabolism on both pathogens remain unknown.

### Modification of Host-Bacterium Interaction

Cooperative behaviors such as protection against antimicrobials and trophic cooperation probably contribute to the establishment and maintenance of *P. aeruginosa* and *S. aureus* co-infections. In addition, interactions between the two pathogens were shown to affect their abilities to colonize host and settle as chronic infections (Figure 4C). Adhesion to host components is an important feature for colonization, and *S. aureus* can attach more efficiently to abiotic surfaces during co-culture with *P. aeruginosa* (Kumar and Ting, 2015). In these conditions, *S. aureus* presented an up-regulation of several proteins involved in adhesion to platelets or to the extracellular matrixes of various hosts, such as serine rich glycoproteins and the Ebh protein. These results suggest that co-culture may increase *S. aureus’s* adhesion to host cells (Kumar and Ting, 2015). In line with this, co-culture with *P. aeruginosa* CF strains was shown to induce the overexpression of nine virulence factors of *S. aureus*. Among them, the Tet38 transporter promoted the internalization of *S. aureus* within epithelial pulmonary cells during coexistence with *P. aeruginosa* (Briaud et al., 2019). Finally, HQNO-mediated induction of the SCV phenotype can increase the intracellular survival of *S. aureus* (Mitchell et al., 2010). Cell internalization and the SCV phenotype, both promoted by *P. aeruginosa*, could thus contribute to the success of *S. aureus* infection.

Limiting the induction and efficiency of immune responses is a strategy already developed by *P. aeruginosa* during its genetic adaptation to the CF environment. Interestingly, *P. aeruginosa-S. aureus* co-infection induces a specific host response different from mono-infections in a rat otitis model (Yadav et al., 2017). Using bacterial supernatants, Chekabab et al. (2015) observed that molecules secreted by *S. aureus* decreased the hosts’ immune response induced by *P. aeruginosa* supernatant. Thus, the protein SpA produced by *S. aureus* was shown to bind either to *P. aeruginosa* Psl exopolysaccharide or to anti-Psl IgG antibodies, protecting *P. aeruginosa* from Psl recognition by neutrophils and thus phagocytosis (Armbruster et al., 2016; Figure 4C). In addition, *S. aureus* presence induces transcriptomic down-regulation of several antigenic factors in *P. aeruginosa*, such as genes involved in secretion and flagellum synthesis (Miller et al., 2017).

Consequently, several studies suggest that *P. aeruginosa* and *S. aureus* co-infection favors chronic infections. Cigana et al. (2018) indeed observed that preliminary colonization by *S. aureus* increased the ability of PA14 and CF-adapted strains to establish a chronic infection in a murine model. Interestingly, such infection kinetics are frequent in CF patients as *S. aureus* is one of the first colonizers in the lungs of young children, whereas *P. aeruginosa* infection occurs upon adolescence (French Cystic Fibrosis Register, 2017). On another note, *S. aureus-P. aeruginosa* co-infections were shown to delay chronic wound healing and thus bacterial clearing (Pastar et al., 2013). Finally, Zhao et al. (2018) observed that mice presented an improved survival when their lungs were infected by a mix of *P. aeruginosa*, *S. aureus* and *Klebsiella pneumoniae*, in comparison to mice infected only with *P. aeruginosa*. Although this effect cannot be specifically attributed to the presence of *S. aureus* and/or *K. pneumoniae*, co-infection appears to reduce the host mortality of *P. aeruginosa* infections and thus promote longer infections. Altogether, these studies suggest that co-infection favors the establishment of chronicity, since the concomitant presence of pathogens will improve their persistence within the infection site (Limoli and Hoffman, 2019).

## *P. aeruginosa* and *S. aureus* Are Not Alone

So far, the adaptive process and interactions of *P. aeruginosa* and *S. aureus* remain those most documented, as their infections are the most prevalent and severe in the context of CF (Machan et al., 1992; Mashburn et al., 2005; Hotterbeekx et al., 2017). However, numerous other microorganisms can colonize the CF environment and are thus susceptible to interact with *P. aeruginosa* (French Cystic Fibrosis Register, 2017). Among them, the fungus *Aspergillus fumigatus* is involved in a nutritional competition with *P. aeruginosa*. In this context, growth, biofilm formation and hyphal structure of the fungus are altered by *P. aeruginosa* secreted factors such as rhamnolipids, phenazines and the QS signals 3OC12-HSL and PQS (O’Brien and Fothergill, 2017; Briard et al., 2019; Sass et al., 2019; Chatterjee et al., 2020). As *P. aeruginosa’s* adaptation to the CF lung environment affects the secretion of these factors, it is likely that its relationship with *A. fumigatus* also evolves toward a coexistence-like interaction allowing the establishment of stable *P. aeruginosa-A. fumigatus* co-infections. Although no direct evidence has been established yet, this hypothesis is supported by the reduced antifungal activity shown for mucoid *P. aeruginosa* isolates (Briard et al., 2019; Chatterjee et al., 2020). Moreover, *P. aeruginosa* infection rather precedes *A. fumigatus* colonization and can even promote aspergillosis (O’Brien and Fothergill, 2017; Briard et al., 2019; Chatterjee et al., 2020). The aggressive behavior of *P. aeruginosa* is also observed toward the yeast *C. albicans* and the bacteria from the *Burkholderia* spp (Fourie et al., 2016; O’Brien and Fothergill, 2017; Fourie and Pohl, 2019; Limoli et al., 2019). However, it appears that these microorganisms are not powerless in front of *P. aeruginosa* and can even take advantage of this interaction within mixed-species biofilm. Antimicrobial resistance and virulence of *C. albicans* and *P. aeruginosa* are thus enhanced during their interaction, particularly through an increased production of phenazines by *P. aeruginosa* and ethanol by *C. albicans* (Fourie et al., 2016; O’Brien and Fothergill, 2017; Fourie and Pohl, 2019; Alam et al., 2020; Bandara et al., 2020; Hùlková et al., 2020; Ibberson and Whiteley, 2020). In the same way, *P. aeruginosa* alginate can protect *B. cenocepacia* from the host immune system (O’Brien and Fothergill, 2017).

If microbial adaptation to the CF environment favors such win-win interactions is yet to be determined. In particular, one might wonder if the increased virulence within mixed-species biofilm with *C. albicans* is a conserved response in CF-adapted *P. aeruginosa*, as the bacterium preferentially evolves toward an avirulent lifestyle (Bianconi et al., 2015; Marvig et al., 2015a; Riquelme et al., 2020). One another note, and as described earlier for *S. aureus*, *P. aeruginosa* is not alone in evolving. Notably, modifications in LPS and siderophore production, as well as a decrease of mucoidy, virulence and biofilm formation, were demonstrated during *B. cenocepacia’s* adaption to the CF environment (Cullen et al., 2015; Maldonado et al., 2016). These alterations are susceptible to affect its interactions with *P. aeruginosa*, and with other co-colonizing microorganisms.

## Concluding Remarks and Perspectives

In addition to the persistence features of microorganisms, their interactions play a key role in their survival within infectious sites. Although poorly described, this “social” aspect is particularly essential in CF lung infections as they gather significant microorganism richness and densities. *P. aeruginosa’s* adaptation to the CF environment appears to drastically impact its microbial interactions, allowing the development of neutralist and even cooperative behaviors with co-colonizing microorganisms such as *S. aureus* or *C. albicans*. This raises questions about the impacts of these microorganisms on *P. aeruginosa’s* adaptation to the CF lung environment: can their presence constitute a selective force and promote the establishment of cooperative interactions?

Such cooperation is indeed an attractive strategy to promote microbial persistence within lungs: (i) it is less costly for both partners as the production of virulence or resistance factors is no longer required, and (ii) it can provide advantageous features to both partners of the interaction. CF-adapted strains of *P. aeruginosa* and *S. aureus* can thus benefit from reciprocal protection against antibiotics and metabolic cooperation, despite strong competition *in vitro* and/or between non-adapted strains.

Ultimately, these cooperative interactions could contribute to the establishment of “Climax communities,” i.e., microbial communities with a steady structure within the CF ecosystem (Quinn et al., 2016). Interestingly, *S. aureus* and *P. aeruginosa* are part of one of these Climax communities *in vivo*, suggesting that their interaction may stabilize and maintain co-infection by these two pathogens. This phenomenon could explain the high proportion of *P. aeruginosa-S. aureus* co-infected patients within CF cohorts (Limoli et al., 2016; Briaud et al., 2020), and more broadly the positive or negative associations observed between different pathogens (Granchelli et al., 2018). Nevertheless, the impacts of *P. aeruginosa* and *S. aureus* co-infections on clinical outcomes remain unclear and poorly described (Limoli et al., 2016; Briaud et al., 2020). Taking into account the nature of their interaction might unveil new aspects of their pathogenesis and their ability to durably persist within CF lungs.

## Author Contributions

KM and LC were primarily responsible for preparing the review. FV and PB contributed to writing the review and editing the final version. All authors contributed to the article and approved the submitted version.

## Conflict of Interest

The authors declare that the research was conducted in the absence of any commercial or financial relationships that could be construed as a potential conflict of interest.
